# Drivers and effects of DRG coding abuse based on hot cheese model: a qualitative study

**DOI:** 10.3389/fpubh.2026.1826650

**Published:** 2026-06-12

**Authors:** Shuo Liu, Chang-Chang Chen, Li Zhao, Zhi-Ping Yang, Dai-Ming Fan, Xue-Jun Hu

**Affiliations:** 1 First Affiliated Hospital of Air Force Medical University, Xi’an, China; 2 Department of Nursing, Air Force Medical University, Xi’an, China; 3 Air Force Medical Training Base, Air Force Medical University, Xi'an, China

**Keywords:** DRG coding abuse, drivers, effects, hot cheese model, qualitative study

## Abstract

**Background:**

The national-level China Healthcare Security Diagnosis-Related Groups (CHS-DRG) reform in China has been underway for 6 years. However, the marginal benefit of controlling medical insurance fund expenditures has been decreasing, mainly due to the emergence of game behaviors among healthcare providers. The study aimed to explore the internal driving factors, effects and their interrelationships of DRG downcoding, upcoding and ambiguous cases to propose the intervention measures based on the hot cheese model.

**Methods:**

This study was conducted through semi-structured interviews with 22 participants from various positions in hospitals in Shaanxi Province, China. The hot cheese model was introduced for qualitative analysis to systematically analyze the manifestations, subjective and objective driving factors, and internal and external effects of DRG coding abuse.

**Results:**

The subjective, objective drivers and effects of DRG coding abuse were revealed, and a hot cheese model was established, consisting of three defensive layers: layers of hospital organization environment, doctor-filled first page of medical records, and medical record/insurance department review.

**Conclusion:**

DRG upcoding should be distinguished between subjective and objective driving factors for targeted intervention. The potential effect of DRG downcoding and ambiguous cases should not be underestimated. Future research should focus on intelligent early warning by natural language processing on pre-coding of medical record texts.

## 1 Background

Diagnosis-Related Groups (DRG), as a risk-adjusted case mix tool, have been promoted and applied globally (1). China launched its national-level DRG reform in 2019, covering all provinces and most inpatient medical institutions by 2025 (1). However, this did not significantly control the expenditure of the national medical insurance fund, and even inpatient expenses increased by 16% year-on-year in 2023 (2, 3). This is mainly due to the game behavior of healthcare providers, which means by adjusting diagnosis or procedure codes, cases were classified into higher-paying groups, enabling the hospital to receive additional compensation, leading to overpayment of the national medical insurance fund (4). In this study, DRG upcoding, downcoding and ambiguous cases were regarded as a risk incident termed as DRG coding abuse, whose manifestations include ICD coding errors, substitution of primary diagnoses, and mismatches among diagnoses, examinations, and treatments (5).

Previous studies mainly focused on upcoding, which is the most common, harmful and well-researched. It had a wide range of macro risk factors, including patients, healthcare markets, operators, DRG control systems, and case-mix systems. And its drivers included stress, attitudes, and vulnerabilities (5, 6). These macro and micro factors were mixed together, making it difficult to precisely identify driver problems and formulate intervention measures at the operational level. In addition to upcoding, the situation where downcoding leads to defrauding medical insurance funds has been underestimated (7).

Current behavioral economics studies on medical behaviors under DRG payment reform have formed a diversified analytical paradigm. Luo et al. (8) applied the Theory of Planned Behavior and confirmed that physicians’ behavioral attitudes, subjective norms, and perceived behavioral control could significantly explain the formation mechanism of strategic medical behaviors. Li et al. (9) sorted out key risk links of upcoding, summarized five core risk dimensions covering the medical market, coding staff, hospitalized patients, DRG case-mix system, and supervision and governance system, and further constructed a risk factor analysis framework for inappropriate upcoding based on the key links of diagnostic and coding information transmission. From the perspective of the fraud triangle theory, Wang et al. (10) pointed out that performance pressure, institutional loopholes, and moral rationalization jointly induce adverse behaviors such as upcoding and patient stay splitting. Based on the principal-agent theory, Fu et al. (11) revealed that while curbing traditional moral hazards, DRG payment reform has triggered new agency problems including service skimping and coding manipulation. Zhang et al. (12) adopted an evolutionary game model and verified that regulatory intensity and penalty stringency dominate the evolutionary stability of medical providers’ non-compliant behaviors. Although the above studies have analyzed adverse medical behaviors from the perspectives of individual cognition, organizational incentives, information transmission, and institutional game, they are generally limited by fragmented dimensional analysis and static attribution. Existing literature lacks integrated interpretation of the cascading transmission of multiple risk factors and the systematic failure of internal defense mechanisms.

This study adopts a theory-driven qualitative research design and extends the discussion from upcoding to downcoding and ambiguous cases. We introduced the hot cheese model (13) as a pre-structured conceptual model to define the “defense layer” in hospital coding-related processes, and used qualitative interviews to explore the manifestations, drivers, effects and interrelationships of DRG coding abuse through thematic analysis. It organizes perceived drivers and defense failures into a layered process model using the Hot Cheese framework, ultimately forming intervention measures for DRG coding abuse.

## 2 Methods

This qualitative study adopts an interpretivist epistemological orientation, focusing on the in-depth interpretation of management and practical issues within specific real-world contexts. Meanwhile, it employs a theory-driven deductive analytical approach, taking the Swiss Cheese Model as the analytical framework. Through thematic analysis, the semi-structured interview data is systematically sorted and thematically refined step by step. Relying on the multi-layered defense dimensions of the Swiss Cheese Model, this study sorts out and integrates various influencing factors of anomalies, and ultimately clarifies the key incentives and internal mechanism logic behind abnormal DRG grouping and payment. This study was designed, conducted, and reported in accordance with the Standards for Reporting Qualitative Research (SRQR). The SRQR checklist is provided in Supplementary file 1 to ensure transparency and methodological rigor.

### 2.1 Conceptual framework

We introduce the hot cheese model to construct a conceptual framework to analyze the coding abuse for CHS-DRG in China. Though the Swiss cheese model proposed by James Reason in 1990 (14), which visualized incidents as holes in cheese slices representing the coincidental overlap of multiple defensive failures and has played a significant role in risk analysis and management of human operation incidents, the Hot Cheese model was introduced by Li et al. (13), to address the former’s limitations, including its static nature, lack of interactivity, and failure to consider the inherent risks within the “defense layer”.

**Figure 1:**
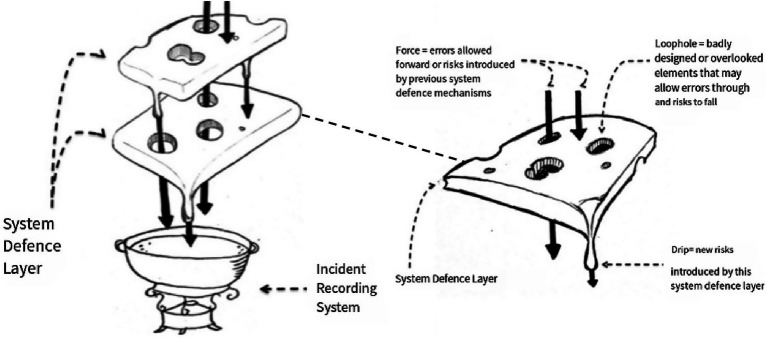
Hot cheese model.

The hot cheese model (see Figure 1), which contained three key factors that lead to accidents. Firstly, “force” referred to the risk of active errors that are allowed to pass, combined with the upstream system defense levels. Secondly, “loophole” referred to a potential situation hidden in a certain layer that ignored the active error of the previous layer, which may lead to force penetration. Thirdly, “drip” was a new risk introduced by the current protective layer due to defects involved. Finally, the cheese that leaks from these defense layers gathers to form a “fondue pot,” indicating the collection of system accident reports for analysis and early warning of the future.

Compared with the classic Swiss cheese model, the hot cheese model can provide a dynamic and proactive interpretation of complex systems. Its greatest innovation is that it can reflect the potential for the defense layer to actively trigger errors (in the medical system, the “drip” error of the defense layer may pose a greater threat because such errors are unexpected and difficult to detect), It views incidents as a combination of active errors and barrier failures, and regards the barrier itself as an active factor (14) that may trigger new errors over time. Overall, it is more suitable than the Swiss cheese model for DRG coding abuse.

### 2.2 Hypotheses of our hot cheese model

Following the research logic of the hospital DRG data generation and flow path, our assumption was that the accumulation of vulnerabilities in various defense layers lead to DRG coding abuse. We identified the key roles and control points of DRG coding abuse, thereby setting the “defense layer” in the hot cheese model: The Hospital Organization Environment layer, the “Doctor-filled First Page of Medical Records” layer, and “Medical Record/ Insurance Department Review” layer. The Hospital Organization Environment layer mainly includes the financial pressure, administrative pressure and performance evaluation mechanism of the hospital/department; the “Doctor-filled First Page of Medical Records” layer involves the diagnosis and treatment behavior, medical record writing norms and the selection of the main diagnosis on the first page; the “Medical Record/Insurance Department Review” focuses on the professional ability of coders and the quality control of the first page, as well as the situation of unreasonable charges and inability to group under DRG.

### 2.3 Design, sample and recruitment

The sample size was determined based on the principle of data saturation in qualitative research. From September 2025 to February 2026, we conducted semi- structured interviews with 22 participants from 5 hospitals’ various positions in Shaanxi Province. All interviewees in this study were hospital-based practitioners, including clinicians, medical coders, hospital medical insurance staff and administrative managers. External medical insurance regulators were not enrolled in the interview sample.

Respondents were selected using purposive sampling to ensure coverage of different departments and job roles, including 12 clinical physicians, 6 medical record coders, 2 personnel from the medical insurance department, and 2 administrative staff from hospital functional departments. The clinical physicians were from orthopedics, gastroenterology, geriatrics, oncology, radiotherapy, cardiology, nephrology and neurology. The exclusion criteria for the research subjects were non-frontline clinical doctors (focusing on scientific research) or respondents who were not familiar with CHS-DRG. Details are provided in Supplementary file 2.

### 2.4 Data collection

After a literature review and five pilot interviews, a semi-structured interview outline was developed based on the hot cheese model and centered around the defense layers in the coding process. Participants were asked about the manifestations of DRG coding abuse, the coding process, problems in the review mechanism, causes, and effects.

All three interviewers in this study possessed a professional background in health management. The research team has long been engaged in studies on medical insurance policies and hospital management. All interviewers received systematic training in semi-structured interviewing skills and ethical norms, with solid practical experience in qualitative fieldwork to ensure standardized data collection. No hierarchical relationships, conflicts of interest, or personal connections existed between interviewers and participants. All interviews were conducted in a neutral and objective manner, with no verbal inducement, subjective hints, or other forms of research bias. As this study was conducted purely for academic purposes, the authenticity and reliability of all interview data were fully guaranteed.

One-on-one interviews were conducted with the research subjects, which lasted between 30 min and 1 h. All interview recordings were transcribed and proofread within 24 h and then analyzed. Data saturation was considered achieved only when no new themes or codes were identified from additional interviews. The interview outline is shown in Supplementary file 3. The study was conducted in accordance with the Declaration of Helsinki and approved by the Institutional Review Board. Written informed consent was obtained from all individual participants prior to the interviews, ensuring their rights to anonymity, voluntary participation, and withdrawal at any time without penalty.

### 2.5 Data analysis

This study adopted theory-driven deductive thematic analysis following the standard procedural framework proposed by Braun and Clarke (15). All interview transcripts were transcribed verbatim and anonymised, with data analysis conducted using NVivo 15.0. Firstly we read through all materials repeatedly to gain full familiarity with the raw data. Guided by the established theoretical model, pre-defined analytical dimensions were applied to conduct initial coding in a top-down, deductive manner. All preliminary codes were further compared, screened, merged and categorised to form sub-themes and core themes. To ensure analytical rigour, two our researchers independently performed dual coding. Any discrepancies in coding were resolved through group discussion and consensus. Finally, all extracted themes were integrated under the theoretical framework to systematically interpret the influencing factors and underlying mechanisms of DRG coding abuse.

## 3 Results

### 3.1 Interview data analysis results

Based on deductive thematic analysis guided by the pre-established conceptual framework (15), all textual interview data were reviewed sentence by sentence and coded systematically to label core viewpoints related to DRG coding abuse. First, all labelled preliminary codes were cross-compared and consolidated, with duplicate and homogeneous entries merged, generating a total of 32 independent concepts and 14 preliminary thematic categories. Second, by integrating the connotation and internal correlation of preliminary categories against the study framework, the 14 initial categories were further refined and condensed into six primary thematic dimensions, including profit and loss of admitted cases, physician-led front-page medical record documentation, hospital organizational context, internal hospital review and supervision, medical insurance fund performance, and differentiated diagnosis-treatment behaviors as well as doctor-patient interactions. Finally, through iterative thematic comparison, correlation screening, and storyline integration, dimensions weakly associated with DRG coding abuse were excluded. The remaining valid themes were logically sorted and summarized, ultimately yielding 4 core themes and 10 sub-themes (Table 1). Full coding details and textual evidence are presented in Supplementary file 4.

**Table 1:** Thematic coding framework table.

No.	Core themes	Sub-themes	Connotation	Typical interview quotes
1	Medical record front sheet filling	Upcoding	Selecting DRG groups with higher weight and payment standard during coding, mainly including incorrect selection of principal diagnosis/surgical procedure or addition of other diagnoses	R11 “For the Cardiac Surgery Department, patent foramen ovale is usually listed as the principal diagnosis if present.” R8 “Previously, the Geriatrics Department would include all diagnoses in the present illness as secondary diagnoses, and elderly patients also request treatment for non-specialty and non-primary diseases during this hospitalization.”
2		Downcoding	Selecting DRG groups with lower weight and payment standard during coding, mainly including incorrect selection of principal diagnosis code or omission of other diagnoses, which is directly related to the experience of physicians and medical record coders	R6 “A friend from another hospital told me they had training saying that if a disease consistently has a low multiple rate, its pricing will be reduced the next year. So we sometimes choose a milder group for patients to be paid by DRG.” R5 “A patient in our Gastroenterology Department died of postoperative infection. I thought the principal diagnosis should be infection, but the Medical Record Department insisted on the original diagnosis, and we had to follow their decision.”
3		Unclassifiable cases	Ambiguous cases (QY category), referring to inconsistent principal diagnosis and principal surgery in the same MDC	R8 “Comorbidity is common in the Geriatrics Department, leading to frequent ambiguous cases, and only one disease can be given primary treatment. “R18” For some complex cases that are not covered by the existing grouping schemes, our medical insurance department will first set them as ambiguous groups and later adopt ‘payment by item’”.
4	Hospital organizational environment	Changes in economic benefits	Changes in the balance of revenue and expenditure of hospitals and departments (i.e., the difference between total revenue and cost expenditure), extended to the average hospital income per treated patient, representing the degree of involutionary competition among hospitals	R18 “Some hospitals developed in an expansionary way with huge early investment and high debt ratio, followed by a single disease spectrum, resulting in the closure.” R22 “Involutionary competition has led hospitals into a vicious circle of ‘increased volume but reduced profit’—the more intense the involution, the lower the weight and the less the income.”
5		Performance accounting mechanism	Whether hospital and department leaders convert case losses into performance wages at a certain ratio when distributing performance wages, thus affecting individual salary levels	R7 “The money lost from treating patients will not be deducted directly from salaries, but it will be converted into performance wage deductions at a certain ratio, which still has an impact.”
6		Administrative pressure from leadership	Verbal and behavioral encouragement or acquiescence of abnormal DRG grouping and payment by hospital, department or section leaders to physicians or medical record coders	R10 “Leaders tell us we cannot overspend and suffer losses, but nor can we spend too little—otherwise the pricing will be lower year by year. We have to calculate the cost while treating patients.”
7	Medical record/insurance department review	Hospital medical record review	The accuracy of coding by hospital medical record coders, including the quantity and experience of the coding team and coding quality	R15 “The Medical Record Department reviews and modifies codes at least three times in the settlement list system.” R14 “The principles for selecting the principal diagnosis on the medical record front page are mostly consistent with those on the settlement list front page, but there are also inconsistencies; the coding error rate of physicians in our hospital is about 12–15%.”
8		Hospital medical insurance supervision	Auditing the quality of the settlement list front page, monitoring unreasonable settlement expenses, and developing an intelligent supervision model for abnormal grouping	R13 “The level of intelligent supervision varies greatly among different hospitals.” R18 The overcoding that leads to overbilling is actually very hard to detect. Generally, only issues such as off-label drug use or duplicate charges based on rules are identified.
9	Impact on medical insurance fund	Overpayment of medical insurance fund	Overpayment of medical insurance fund caused by upcoding and downcoding	R18 “Among the overpayments of medical insurance funds reported by the higher authorities each year, some are questioned by us, but it is very difficult to communicate and verify.” R16 “The situation of Overpayment caused by downcoding is relatively concealed and may be related to cases with low payment ratios.”
10		Fines for verified up-coding	National and local medical insurance bureaus impose fines on hospitals for verified up-coding (direct or indirect), resulting in reduced economic benefits of hospitals	R21 “Our hospital just underwent a medical insurance unannounced inspection a while ago, with only a few days’ notice, which was very sudden.” R20 “The medical insurance inspection team came to conduct an investigation with the task of setting a minimum amount. Our hospital has been making a profit in the past, but it is expected to incur a loss this year.”

### 3.2 Hot cheese model embeddings results

**Figure 2:**
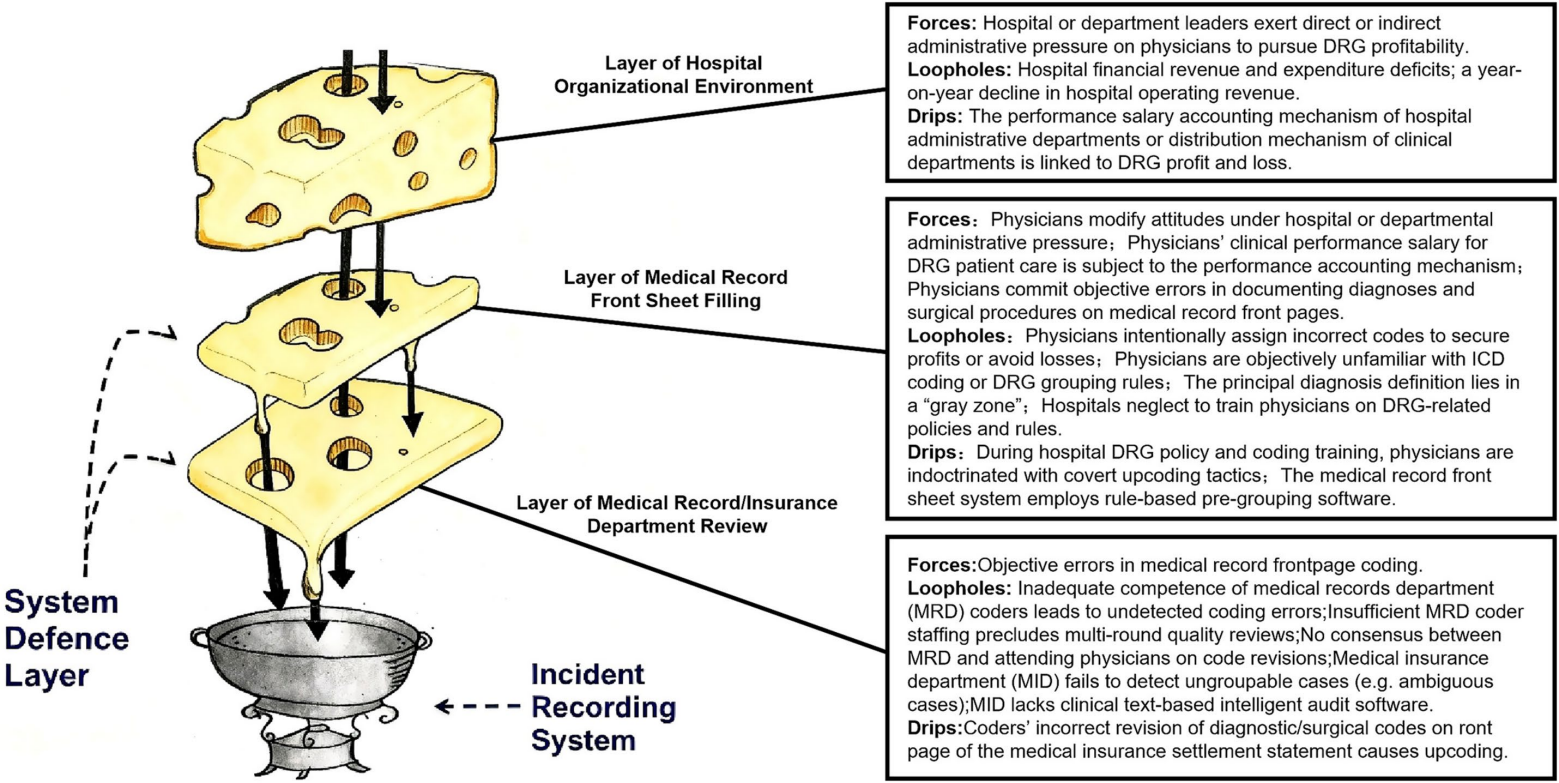
Hot cheese model of DRG coding abuse.

As shown in Figure 2, this paper constructed a hot cheese model of DRG coding abuse, setting up three layers of cheese defense from the perspective of the hospital as a whole and the DRG data flow process.

#### 3.2.1 Layer of hospital organizational environment

The hospital organizational environment set as the first line of defense, with economic pressure, administrative orientation, and performance orientation forming the initial holes of the hot cheese model. The salary system in Chinese hospitals is different from the annual salary system in high-income countries, with a two-tier distribution of performance-based pay linked to hospital income. When the income and expenditure balance of hospitals and departments is under continuous pressure, leaders may induce upcoding through administrative pressure or tacit approval. The performance assessment mechanism formulated by the hospital’s health economics department or the performance-based salary redistributed by department leaders will be linked to the DRG loss. In this environment, the distorted incentive orientation causes doctors’ attitudes and behaviors to mutate, laying the groundwork for subsequent layers of failure.

#### 3.2.2 Layer of medical record front sheet filling

The second line of defense is the Layer of medical record front sheet filling. Under the double pressure of performance-based salary and administration, when doctors fill in the names of diagnoses/surgeries on medical record front sheet filling, some subjectively tend to choose coding strategies that are beneficial to revenue and fabricate primary or secondary diagnoses. Some objectively do not master the coding rules of DRG and ICD, resulting in upcoding or downcoding. Several diseases are in the gray area between the National Health Commission’s “Quality Specification for Filling in Inpatient Medical Record Front Sheet” and the National Healthcare Security Administration’s “Specification for Filling in Medical Insurance Fund Settlement List” rules, making it difficult to define the primary diagnosis and resulting in incorrect selection. As a result, hospitals provided DRG-related training for doctors, but unfortunately, this can also become a “drip” that is wrongly instilled with concealed high-set techniques.

#### 3.2.3 Layer of medical record/insurance department review

The third line of defense is the medical record/insurance review layer. In the face of errors such as “main diagnosis/surgery error”, “too many other diagnoses”, and “missing diagnosis” that appear on the first page filled in by doctors, it may be difficult to completely correct the deviations entered by the front-end doctors due to the personal ability of the medical record coders, the imperfect multiple review mechanism of the coding team, communication compensation between coders and doctors, etc. What’s more serious is that some hospital medical record departments may modify the originally correct code on the first page of the settlement list to make a profit, which is also a “drip” in this defense layer. When the health insurance regulatory system fails to identify these errors in time, the layers of defense of cheese are breached one after another, eventually leading to frequent DRG coding abuse, the accumulation and outbreak of health insurance payment risks, and eventually forming the “cheese hotpot” of abnormal event records for study.

### 3.3 Interactions of the drivers and effects

**Figure 3:**
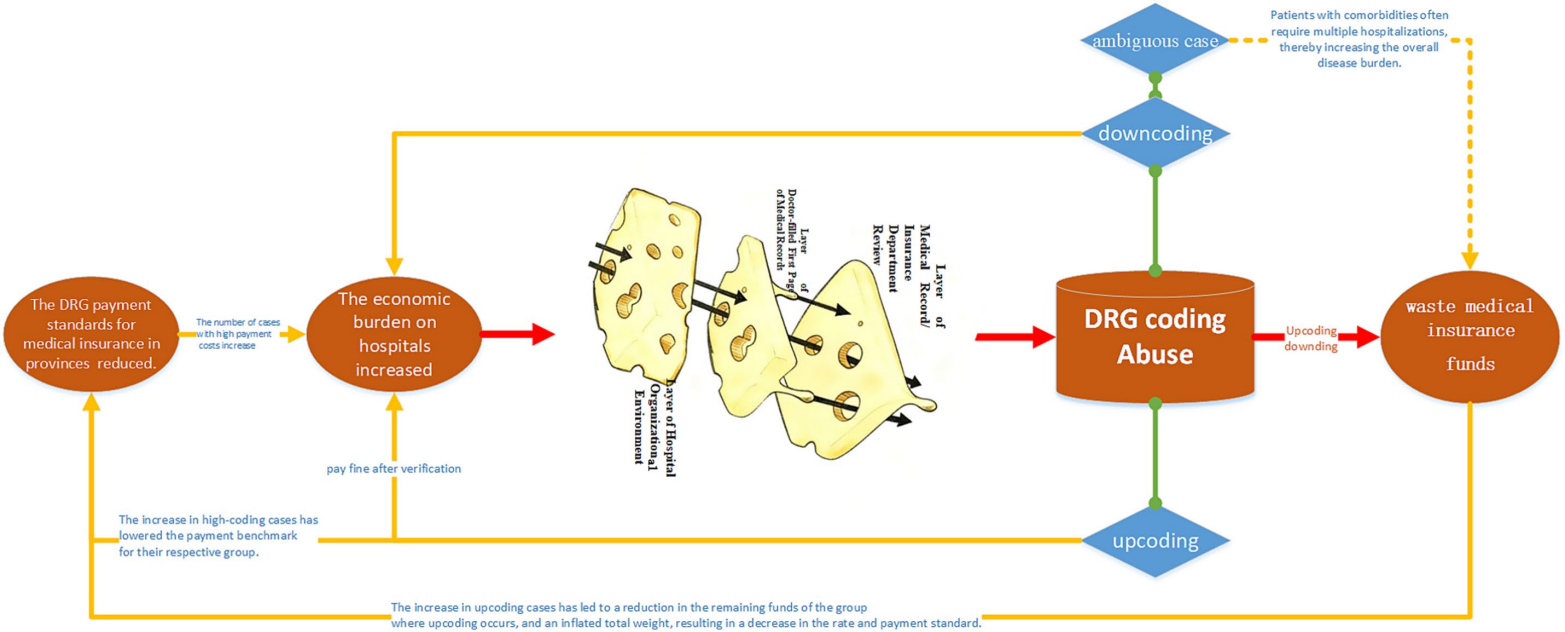
Diagram of interactions of the drivers and effects in DRG coding abuse.

Figure 3 illustrates plausible correlations between drivers and effects associated with DRG coding abuse, indicating that upcoding could generate notable and long-term adverse consequences, while downcoding and ambiguous coding cases also exert non-negligible influences.

Firstly, although upcoding will bring more revenue to the hospital immediately, once discovered, it will be subject to huge fines and administrative penalties. As quoted from R20 “The medical insurance inspection team came to conduct an investigation with the task of setting a minimum amount. Our hospital has been making a profit in the past, but it is expected to incur a loss this year.” This could increase financial pressure on hospitals, with downcoding presenting a similar situation.

Secondly, miscategorization of upcoding cases into mismatched DRG groups may distort the authentic cost profile of relevant disease groups. As quoted from R10 “Leaders tell us we cannot overspend and suffer losses, but nor can we spend too little—otherwise the pricing (payment standard) will be lower year by year. Nevertheless, the payment standard was still lowered in the subsequent year, which may be partially attributed to the distorted group costs caused by DRG coding error in other medical institutions.” Specifically, given that upcoding is primarily driven by financial incentives, such cases may gradually reduce the payment benchmark of the corresponding DRG group.

At the same time, the accumulation of upcoding may inflate the overall weight of regional disease groups, trigger a reduction in annual payment rate and DRG group payment standard, and disrupt the allocation of medical insurance funds distributed by case weights across different hospitals. As quoted from R22 “Involutionary competition has led hospitals into a vicious circle of ‘increased volume but reduced profit’—the more intense the involution, the lower the weight and the less the income.”

## 4 Discussion

The DRG governance models in Western countries are fundamentally shaped by their distinct medical insurance systems. The United States adopts a market-dominated commercial health insurance system, with only public insurance programmes (e.g., Medicare) applying unified DRG grouping standards, while private insurers formulate payment rules independently (16). European countries rely on statutory social health insurance, where DRG frameworks are jointly formulated through multi-party negotiation among medical associations, insurance institutions and regional authorities under sound legal and professional supervision (17). Generally, both American and European DRG systems feature decentralized governance, rule-based operation and high institutional autonomy of medical institutions. There is no vertical hierarchical administrative control jointly implemented by health and insurance authorities, nor a top-down performance appraisal system that tightly links DRG outcomes to departmental or individual physician compensation. Supervision in these contexts mainly depends on legal constraints, third-party independent audits and ex-post compliance reviews, rather than routine penetrating administrative inspection. Accordingly, DRG coding abuse in Western healthcare settings—though they include strategic upcoding driven by financial incentives—typically arise as decentralized, institution-level behaviors, rather than as systematically cascading deviations under hierarchical administrative pressure (18).

In contrast, China’s DRG reform is advanced under a publicly funded basic medical insurance system with strong administrative leadership and hierarchical governance (19). A vertical transmission mechanism has been established as insurance regulation-hospital indicator decomposition-departmental assessment-individual clinical implementation. The mandatory bundling of DRG performance appraisal with departmental income and personal salary serves as a core incentive constraint in domestic hospitals (20). Driven by superimposed administrative assessment pressure and economic benefit incentives, DRG coding abuse in China have evolved from simple technical mistakes into systematic, dynamic and multi-level coupled deviations (19, 20). The Hot Cheese Model focuses on dynamic interaction of institutional vulnerabilities, progressive erosion of internal control lines and multi-factor cascading risks, which delivers higher contextual adaptability and explanatory power for systematic DRG payment anomalies in the Chinese institutional context.

Previous studies mainly focused on three key roles “operators, managers, and regulators” (21, 22). We believe this framework is rather macroscopic: The concept of “management” is overly broad, as it neither articulated the internal role division within hospitals nor emphasized the critical functions of specialized departments such as the medical insurance department and the health economics department. Furthermore, the role of regulators has not been well reflected. It is suggested that apart from external regulators, there are also departments within the hospital that undertake the functions of regulators. In addition, they had not systematically explored the interaction between drivers and effects. Both qualitative and quantitative studies have only conducted static structural analyses such as universality, type, and risk characteristics, and have not proposed actionable interventions for hospital DRG coding processes from the perspective of dynamic management trajectories.

### 4.1 Objective and subjective drivers and intervention

Our research suggests that the drivers of DRG coding abuse included both subjective and objective aspects. The subjective drivers referred to doctors deliberately choosing diagnostic codes that were favorable for higher payments under the drive of profit, and still making biased reports knowing that they did not conform to clinical practice. The objective driver was due to incomplete mastery or misunderstanding of the DRG grouping rules and ICD coding standards, resulting in unconscious incorrect DRG coding. Although they have different motives, both are manifested as superimposed penetration of defense layer vulnerabilities in the hot cheese model. They shield each other in an environment of information asymmetry, increasing the difficulty of regulatory identification and ultimately having a profound effect on medical insurance funds, hospital economic benefits, and medical practices.

Objectively driven DRG coding abuse refer to upcoding/downcoding caused by incomplete mastery or misunderstanding of the rule standards. Its main manifestations were as follows: (1) coding errors referred to a mismatch with the ICD coding standards; (2) misspecification are defined as no corresponding treatment or positive test result; (3) resequencing (21) of primary and secondary diagnoses/surgeries was often due to the logical mismatch between doctors’ clinical thinking and DRG coding rules, and was closely related to the differences from treatment decisions and primary selection of complex diseases; (4) omission of diagnosis and/or surgery names was directly related to the doctors’ experience and was often magnified due to the complexity of the clinical pathway and the abundance of comorbidities, especially in cases of patient interdepartmental transfer or multidisciplinary treatment; and (5) diagnosis and surgical names across major diagnostic categories, referred to as the ambiguous case.

For objective drivers, the study suggest that the key steps of its intervention are in the “doctor fill the first page layer” and the “medical record/medical insurance review layer”. At the pre-event level, doctors need to know sufficient coding rules to fill in the home page correctly, and coders need sufficient clinical knowledge to reverse examine the diagnosis and treatment norms. It is recommended to strengthen the two-way training of doctors and medical record coders and improve the quantity and quality of the coder team; At the intermediate level, it is recommended to promote the establishment of a coding quality evaluation system within hospitals, incorporate coding accuracy into departmental performance assessment, and strengthen the “clinical department - medical record coding” collaborative review mechanism; Develop an intelligent early warning model system (23) for DRG coding abuse to achieve error prediction and real-time correction, reducing the occurrence of objective upcoding from the source. At the post-event level, medical records and medical insurance departments should improve the data feedback mechanism, regularly inform doctors of abnormal coding situations and cause analysis, implement closed-loop management to reduce the space for upcoding/downcoding to breed, and enhance the overall robustness of DRG assignment and payment.

Subjectively driven DRG coding abuse refers to defrauding the medical insurance fund caused by deliberately choosing upcoding or downcoding under the drive of interests (24). Among them, defrauding caused by downcoding is more concealed. It usually occurs when the payment ratio of a certain case in its corresponding DRG group is low, in the form of “replacing the main diagnosis/surgery with a lower weight” or “reducing other diagnoses” in order to obtain a higher payment amount than the per-item payment.

For subjective drivers, the key steps of the intervention are at the “hospital organizational environment layer” and the “doctor fill the homepage layer” based on the hot cheese model. First, it is necessary to pay attention to correcting the attitude of hospital and department leaders towards high pretenses, fully realizing that “high pretenses get away with it for a while, but once discovered, they will pay a huge price”, and avoid exerting administrative pressure on hospital functional agencies and doctors; The DRG performance-based pay calculation and distribution mechanism determines the original motivation of doctors’ subjective upcoding. Hospital authorities (generally led by the department of health economics) and department leaders need to be subject to corresponding supervision to eliminate the wrong incentive orientation of linking performance-based calculation and distribution to personal income. In addition, be cautious of the “embezzlement” style of laissez-faire or even active errors by the medical records department and the medical insurance department. When training doctors on DRG policies and coding rules, it is strictly forbidden to instill hidden upcoding techniques. Last but not least, with the development of national health insurance legalization, doctors, as the starting point of coding, need to first establish an upcoding legal concept. The Health Insurance Distribution and Accountability Act (HIPAA) of the United States clearly defines the term “should know” as “intentionally ignoring or disregarding the truth or false circumstances” and “not needing to prove specific fraudulent intent”. In addition to civil fines, offenders may be sentenced to up to 10 years in prison, and hospitals are barred from participating in health insurance plans (25).

### 4.2 Upcoding, downcoding and ambiguous cases

Keith et al. (26) argued that although “upcoding” is generally defined as “charging for items of a higher complexity level for a higher complexity level for services delivered or documented”, the behavioral mechanisms of upcoding have changed dramatically over the past four decades. The authors highlighted the difficulty in formulating a unified definition of upcoding across diverse healthcare services, which stems from the confusion between complexity and inefficiency in healthcare delivery. For instance, it is challenging to distinguish between “overdiagnosis or overtreatment” and “inappropriate complexity documentation”. This dilemma was prominently reflected in physician interviews, where respondents focused more on the impacts of DRG mechanisms on inefficient practices such as clinical decision-making, admission and discharge arrangements, and patient referrals.

Furthermore, Keith et al. indicated that although hospitals, healthcare providers and clinical coders are highly aware of the adverse consequences of improper coding, inappropriate coding behaviors may still occur when financial incentives are sufficiently strong (26). Similarly, Jürges et al. (27) documented a growing prevalence of artificial manipulation of neonatal birth weights around reimbursement-related thresholds following the implementation of DRG-based payment in Germany’s neonatal care sector, demonstrating a systematic correlation with economic incentives. Such findings are consistent with our interview results, wherein changes in economic benefits were identified as one of the initial driving factors in the cheese hole model. In addition, Harrington et al. categorized upcoding behaviors into intentional and unintentional/negligent patterns. Specifically, intentional upcoding is regarded as fraud, which may result in severe financial penalties or criminal prosecution, whereas unintentional misconduct only leads to the recovery of overpaid funds (28). Correspondingly, this study conducts discussions from the dimensions of subjective and objective influencing factors.

However, substantial heterogeneity exists in the mechanisms underlying downcoding and upcoding. This distinction has long been overlooked in conventional risk frameworks yet is critical for improving the explanatory power of the optimized cheese hole model. Zafirah et al. (29) conducted a medical record audit in teaching hospitals within Malaysia’s MY-DRG system and found that 52.1% (160/307) of cases were assigned lower hospital tariffs, resulting in potential revenue losses for healthcare institutions. Saizan et al. (30) further verified that non-standard clinical documentation and coding operational errors constitute the primary contributors to undercoding. Collectively, it can be inferred that undercoding predominantly arises from passive, systematic and procedural deficiencies, while upcoding is largely driven by proactive strategic responses of stakeholders to external incentives and institutional pressures. The attribute differences between these two-way coding biases refine the multi-layered vulnerability logic of the cheese hole model, and further reveal that distinct types of coding non-compliance correspond to differentiated internal control weaknesses throughout the coding governance chain.

This paper suggested that the effect of downcoding has been underestimated in comparison with upcoding. On the one hand, as the costs of drugs and consumables in centralized procurement have dropped sharply, the proportion of low-rate cases in disease groups with a large proportion of drug and consumables has increased significantly, but the proportion of medical services has not increased in a timely manner. The pay-per-item model does not fully reflect the cost-value, which provides the original motivation for defrauding. On the other hand, even if downcoding does not directly affect the loss of regional medical insurance fund, its accumulation reduces hospital income and increases the economic burden of hospitals, looping back to our hot cheese model constructed in this study.

In addition, ambiguous cases, where the principal diagnosis and the primary surgery do not fall under the same Major Diagnostic Category, should not be overlooked in terms of significance about hindering the trend of multidisciplinary treatment. Though CHS-DRG is inherently designed to accommodate comorbidities and complications, it still struggled to cope with the simultaneous management of two or more equally critical diseases, particularly for ambiguous cases. As quoted from R8 “Comorbidity is common in the Geriatrics Department, leading to frequent ambiguous cases, and only one disease can be given primary treatment”, ambiguous cases may lower the cost of individual treatments, however, they could elevate the overall disease burden concerning patient recovery.

### 4.3 DRG coding abuse affects broader public health

Tough quantitative empirical evidence that directly confirms the relationships between DRG coding abuse and adverse public health outcomes remains limited at present, this paper further explored that how DRG coding abuse impedes the attainment of public health goals from multiple dimensions. At the macro institutional level, the most direct consequence of non-compliant coding is the reduced efficiency of medical insurance fund utilization. Medical institutions can obtain excessive medical insurance reimbursement through inappropriate upcoding without providing corresponding high-quality medical services, thereby causing unnecessary loss of medical insurance funds and waste of public resources. As a core indicator for fund allocation and resource accounting under the prospective payment system, DRG case weights objectively reflect the relative consumption of medical resources. However, selective upcoding inflates the overall weight value and leads to distorted accounting results, which disrupts the rational distribution of medical insurance funds among hospitals and seriously impairs the equity of medical resource allocation. Furthermore, widespread and persistent coding abuse systematically disconnects DRG grouping weights from the actual resource consumption of diseases, making payment standards inconsistent with real clinical costs and ultimately undermining the long-term stability and sustainability of the DRG payment system.

At the micro service level, driven by profit-seeking incentives, hospitals tend to prioritize patients with easily upcoded conditions and evade severe and complex cases, which greatly reduces the accessibility and suitability of essential medical services. In addition, strategic clinical behaviors induced by DRG reimbursement impair overall care quality and integrity. The prevailing phenomenon of “calculating benefits before treatment” makes clinical decisions and treatment plans overly dependent on cost–benefit trade-offs rather than standardized clinical practice. Some facilities fabricate complications or upgrade diagnosis codes to conduct unnecessary examinations and treatments, increasing patient health risks, while others omit essential medical procedures or implement early discharge to control operational costs, compromising the integrity of clinical care and long-term patient prognosis.

From the perspective of social governance, the adverse effects of coding abuse are long-term and spillover. Such non-compliant behaviors aggravate mutual distrust among hospitals, medical insurance authorities and patients, force regulators to strengthen strict review and sanction measures, increase overall regulatory costs, and continuously erode the public credibility and governance capacity of the healthcare system. In conclusion, DRG coding abuse goes beyond simple payment fraud and have become a critical risk hindering the advancement of DRG reform and the realization of comprehensive public health objectives.

### 4.4 Limitations

This study focuses on the China’s situation, which may limit the cross-national generalizability of its findings. Several limitations should be acknowledged in the present research. First, the findings are based on a relatively small, province-specific sample and may not be transferable across regions or payment contexts. Second, the study relies on self-reported perceptions and experiences, which may be shaped by recall bias, strategic framing, or social desirability. Third, the analysis is hospital-centered and appears to underrepresent external regulator perspectives. In addition, the study does not include triangulation with administrative or claims data, which limits the ability to substantiate broader system-level claims. Finally, the conceptual model is promising, but it remains interpretive and exploratory rather than validated.

Moreover, the research scope is confined to the impacts on medical insurance funds and hospital financial performance. As suggested by interview narratives, DRG coding abuse may also interfere with clinicians’ Behavior, e.g., admission and discharge decision-making (4). These underdiscussed adverse outcomes warrant more in-depth investigation in future studies.

## 5 Conclusion

In conclusion, our study suggested that integrating DRG coding abuse with the hot cheese model revealed the subjective and objective drivers, effects and their interrelationships underlying DRG upcoding, downcoding, and ambiguous cases. Furthermore, we have established our hot cheese model for DRG coding abuse, which is composed of three defense layers: layer of hospital organizational environment, medical record front sheet filling and medical record/insurance review. Finally, this study developed targeted interventions for the subjective and objective driving factors of each defense layer in the model, so as to plug the potential holes in each layer and block the transmission of the accident chain.

## Data Availability

The datasets presented in this article are not readily available because the qualitative datasets generated and analyzed during the current study are not publicly available due to ethical and confidentiality restrictions, but parts are available from the corresponding author on reasonable request. Some can be downloaded from the Appendix. Requests to access the datasets should be directed to SL, mickyshuo@126.com.
