# Supplementary material for: Drivers and effects of DRG coding abuse based on hot cheese model: a qualitative study
Source: Front Public Health. 2026 Jun 12;14:1826650. doi: 10.3389/fpubh.2026.1826650 (PMC13303593; doi:10.3389/fpubh.2026.1826650)
Supplement: Supplementary file 3 [file Supplementary_file_3.docx]

**Table S1 Core and Subcategories of Selective Coding with related data**

| **No.** | **Core Category** | **Subcategory** | **Connotation** | **Mentioned Frequency** | **Interview Excerpts** |
| --- | --- | --- | --- | --- | --- |
| 1 | Medical Record Front Sheet Filling | Upcoding | Selecting DRG groups with higher weight and payment standard during coding, mainly including incorrect selection of principal diagnosis/ surgical procedure or addition of other diagnoses | 7 | R11"For the Cardiac Surgery Department, patent foramen ovale is usually listed as the principal diagnosis if present."  R8 "Previously, the Geriatrics Department would include all diagnoses in the present illness as secondary diagnoses, and elderly patients also request treatment for non-specialty and non-primary diseases during this hospitalization." |
| 2 |  | Downcoding | Selecting DRG groups with lower weight and payment standard during coding, mainly including incorrect selection of principal diagnosis code or omission of other diagnoses, which is directly related to the experience of physicians and medical record coders | 15 | R6"A friend from another hospital told me they had training saying that if a disease consistently has a low multiple rate, its pricing will be reduced the next year. So we sometimes choose a milder group for patients to be paid by DRG."  R5 "A patient in our Gastroenterology Department died of postoperative infection. I thought the principal diagnosis should be infection, but the Medical Record Department insisted on the original diagnosis, and we had to follow their decision." |
| 3 |  | Unclassifiable cases | Ambiguous cases (QY category), referring to inconsistent principal diagnosis and principal surgery in the same MDC | 5 | R8"Comorbidity is common in the Geriatrics Department, leading to frequent ambiguous cases, and only one disease can be given primary treatment."R18”For some complex cases that are not covered by the existing grouping schemes, our medical insurance department will first set them as ambiguous groups and later adopt ‘payment by item’". |
| 4 | Hospital Organizational Environment | Changes in economic benefits | Changes in the balance of revenue and expenditure of hospitals and departments (i.e., the difference between total revenue and cost expenditure), extended to the average hospital income per treated patient, representing the degree of involutionary competition among hospitals | 18 | R18"Some hospitals developed in an expansionary way with huge early investment and high debt ratio, followed by a single disease spectrum, resulting in the closure of many. “  R22“Involutionary competition has led hospitals into a vicious circle of 'increased volume but reduced profit'—the more intense the involution, the lower the weight and the less the income." |
| 5 |  | Performance accounting mechanism | Whether hospital and department leaders convert case losses into performance wages at a certain ratio when distributing performance wages, thus affecting individual salary levels | 12 | R7"The money lost from treating patients will not be deducted directly from salaries, but it will be converted into performance wage deductions at a certain ratio, which still has an impact." |
| 6 |  | Administrative pressure from leadership | Verbal and behavioral encouragement or acquiescence of abnormal DRG grouping and payment by hospital, department or section leaders to physicians or medical record coders | 8 | R10"Leaders tell us we cannot overspend and suffer losses, but nor can we spend too little—otherwise the pricing will be lower year by year. We have to calculate the cost while treating patients." |
| 7 | Medical Record/Insurance Department Review | Hospital medical record review | The accuracy of coding by hospital medical record coders, including the quantity and experience of the coding team and coding quality | 6 | R15"The Medical Record Department reviews and modifies codes at least three times in the settlement list system."  R14"The principles for selecting the principal diagnosis on the medical record front page are mostly consistent with those on the settlement list front page, but there are also inconsistencies; the coding error rate of physicians in our hospital is about 12%-15%." |
| 8 |  | Hospital medical insurance supervision | Auditing the quality of the settlement list front page, monitoring unreasonable settlement expenses, and developing an intelligent supervision model for abnormal grouping | 2 | R13”The level of intelligent supervision varies greatly among different hospitals. “  R18The overcoding that leads to overbilling is actually very hard to detect. Generally, only issues such as off-label drug use or duplicate charges based on rules are identified. |
| 9 | Impact on Medical Insurance Fund | Overpayment of medical insurance fund | Overpayment of medical insurance fund caused by upcoding and downcoding | 3 | R18"Among the overpayments of medical insurance funds reported by the higher authorities each year, some are questioned by us, but it is very difficult to communicate and verify."  R16"The situation of Overpayment caused by downcoding is relatively concealed and may be related to cases with low payment ratios." |
| 10 |  | Fines for verified up-coding | National and local medical insurance bureaus impose fines on hospitals for verified up-coding (direct or indirect), resulting in reduced economic benefits of hospitals | 2 | R21"Our hospital just underwent a medical insurance unannounced inspection a while ago, with only a few days' notice, which was very sudden."  R20 "The medical insurance inspection team came to conduct an investigation with the task of setting a minimum amount. Our hospital has been making a profit in the past, but it is expected to incur a loss this year." |
