# Supplementary material for: Drivers and effects of DRG coding abuse based on hot cheese model: a qualitative study
Source: Front Public Health. 2026 Jun 12;14:1826650. doi: 10.3389/fpubh.2026.1826650 (PMC13303593; doi:10.3389/fpubh.2026.1826650)
Supplement: Supplementary file 4 [file Supplementary_file_4.docx]

**Supplementary Material: SRQR Compliance Statement of “Drivers and Effects of DRG Coding Abuse based on Hot Cheese Model:A Qualitative Study”**

This study was reported in full accordance with the 21-item Standards for Reporting Qualitative Research (SRQR) checklist. All items related to study design, setting, participants, data collection, data analysis, trustworthiness, reflexivity, ethics, results, discussion, and implications have been adequately addressed in the manuscript.Standards for Reporting Qualitative Research (SRQR)

**21-Item Checklist**

**Reference**:

O’Brien BC, Harris IB, Beckman TJ, Reed DA, Cook DA. Standards for Reporting Qualitative Research: A Synthesis of Recommendations. *Am J Epidemiol*. 2014;180(10):1005–1012.

| **No.** | **Domain** | **SRQR Item** | **Yes** | **No** | **Not applicable** |
| --- | --- | --- | --- | --- | --- |
| 1 | Title & Abstract | The title and abstract clearly indicate the qualitative nature of the study. | ☑ | □ | □ |
| 2 | Introduction & Context | The research problem, purpose, and research questions are clearly stated. | ☑ | □ | □ |
| 3 | Introduction & Context | Relevant theoretical or conceptual framework is described. | ☑ | □ | □ |
| 4 | Methods | Qualitative study design is identified and justified. | ☑ | □ | □ |
| 5 | Methods | Study setting and location are described. | ☑ | □ | □ |
| 6 | Methods | Participants and sampling strategy are described. | ☑ | □ | □ |
| 7 | Methods | Data collection methods (e.g., interviews, observations) are described. | ☑ | □ | □ |
| 8 | Methods | Procedures for data collection (e.g., interview guide, pilot testing) are described. | ☑ | □ | □ |
| 9 | Methods | Procedures for ensuring data quality and trustworthiness are described. | ☑ | □ | □ |
| 10 | Methods | Data saturation is addressed. | ☑ | □ | □ |
| 11 | Methods | Data analysis procedures (e.g., coding, thematic analysis) are described. | ☑ | □ | □ |
| 12 | Methods | Reflexivity (researcher’s role and potential bias) is addressed. | ☑ | □ | □ |
| 13 | Methods | Ethical approval and informed consent are reported. | ☑ | □ | □ |
| 14 | Results | Findings are supported by data (e.g., participant quotations). | ☑ | □ | □ |
| 15 | Results | Findings are presented logically and coherently. | ☑ | □ | □ |
| 16 | Results | Key themes or categories are clearly identified. | ☑ | □ | □ |
| 17 | Results | Findings are directly linked to the research questions. | ☑ | □ | □ |
| 18 | Discussion | Findings are interpreted in the context of existing literature. | ☑ | □ | □ |
| 19 | Discussion | Limitations of the study are acknowledged. | ☑ | □ | □ |
| 20 | Other | Implications for practice, policy, or future research are stated. | ☑ | □ | □ |
| 21 | Other | Conclusions are supported by the study findings. | ☑ | □ | □ |
